# Upcycling of Waste Durian Peel into Valued Fe/N Co-Doped Porous Materials as Peroxymonosulfate Activator for Terramycin Oxidation

**DOI:** 10.3390/molecules30051005

**Published:** 2025-02-21

**Authors:** Kewang Zheng, Rui Liu, Lihang Shen, Wei Li, Caiqin Qin

**Affiliations:** 1 School of Chemistry and Materials Science, Hubei Engineering University, Xiaogan 432000, China; 2 Faculty of Engineering and Computing, The University of Sydney, Sydney, NSW 2050, Australia

**Keywords:** durian peel, cellulose, degradation, peroxymonosulfate

## Abstract

Nitrogen-doped graphene-coated Fe nanoparticles (EC@N_6_Fe_0.6_-700) were synthesized through the pyrolysis of a durian peel-supported urea ferric salt mixture. These materials were subsequently utilized to activate peroxymonosulfate (PMS) for oxidation of terramycin (TEC). The incorporation of an optimal amount of urea and ferric nitrate during the synthesis of materials significantly improves the catalytic activity of the resulting catalysts after pyrolysis. Using EC@N_6_Fe_0.6_-700 catalyst at a concentration of 0.10 g L^−1^, 98.55% oxidation of 20 mg L^−1^ TEC is achieved within 60 min. Additionally, EC@N_6_Fe_0.6_-700 exhibits exceptionally low metal leaching, with levels remaining below 0.25 mg L^−1^. The EC@N_6_Fe_0.6_-700 shows remarkable stability during oxidation and effectively resists interference, reusability, and robust stability throughout the oxidation process. The mechanism of the EC@N_6_Fe_0.6_-700/PMS/TEC system is determined, and the ^1^O_2_ is the main reactive oxygen species (ROSs). The XPS analysis confirms that the primary active sites are Fe^0^, as well as nitrogen-doped regions within the carbon matrix. This research demonstrates that by integrating iron and nitrogen with durian peel, it is possible to develop a PMS activator with satisfactory oxidation performance for the degradation of environmental pollutants.

## 1. Introduction

Water scarcity has become one of the greatest challenges of this century due to population growth, climate change, and worsening water pollution. Water pollution sources can be divided into industrial pollution, agricultural pollution, and domestic pollution [1,2]. Among them, industrial pollution is particularly harmful. As an important antibiotic, Terramycin (TEC) was widely used in various fields such as feed, biology, medicine, and aquaculture [3]. Nevertheless, the limited utilization of living organisms and the large amount of excess effluent discharge have led to frequent detection of TEC and its derivatives in nature, causing serious ecological hazards [4]. Results indicated that TEC not only inhibited the growth rate of algae but also significantly affected the embryonic lethality of crustaceans and zebrafish [5]. In addition, it was also harmful to human health and could cause many diseases. Consequently, the development of effective methodologies for the oxidation of TEC from water is imperative for the preservation of ecological balance.

The removal methods of antibiotics in water include adsorption, biodegradation, and advanced oxidation processes (AOPs) [6]. AOPs have attracted widespread attention to oxidize antibiotics due to their strong oxidizing ability and operational simplicity [7]. Compared to other AOPs, the AOP based on SO_4_^•−^ has many advantages, such as wider pH tolerance, longer half-life, and higher redox potential [8]. Generally, SO_4_^•−^ can be produced by activating PMS, while strategies of heating, ultraviolet, and chemical materials can be used to activate PMS [9,10]. Among these methods, PMS activation systems that utilize heterogeneous catalysts have garnered significant attention due to their satisfactory oxidation activity and cost-effectiveness [11,12]. In the field of transition metal research, Fe-based materials, including Fe^0^, Fe_3_C, and Fe_3_O_4_, have demonstrated significant potential in the activation of PMS for environmental remediation. These materials offer several advantages, including non-toxicity, a wide range of sources, eco-friendliness, and cost-effectiveness, making them promising candidates for further study [13,14]. However, Fe-based materials activating PMS have some disadvantages, such as excessive Fe^2+^ quenches the SO_4_^•−^ produced, the existence of excessive Fe^3+^ can consume PMS, leading to the lower PMS utilization efficiency and the Fe nanoparticles were easily aggregated together [10]. On the other hand, due to their good biocompatibility, abundant source, low cost, and stability, carbon-based materials have garnered significant attention as an activator for PMS to degrade organic pollutants [15]. But non-metallic carbon-based materials have the disadvantage of insufficient catalytic activity. However, carbon-based materials exhibit limitations in terms of their oxidation activity. To overcome these disadvantages, some strategies have been applied, such as a combination of carbon material and metal material to generate co-doped composite nanomaterials. Studies have indicated that transition metal and heteroatom (B, N, P, S, etc.) co-doped carbon-based nanomaterials can not only effectivity enhance their catalytic performance but also decrease the leaching amount of metal and aggregation [16]. Although the commonly used carbon materials, including carbon nanotubes, fullerene, and graphene nano-diamonds, have good performance in activating PMS, they have high costs, ecotoxic properties, and challenging scalability. Hence, it was meaningful to produce carbon catalysts by using abundant and inexpensive biomass waste precursors [17].

Durian (Durio zibethinus) is cultivated extensively in Southeast Asian countries, as well as in the Chinese provinces of Guangdong and Hainan [18]. This is due to its high nutritional, medicinal, and economic value. However, it is noteworthy that the flesh of the fruit typically accounts for a mere 20–30% of the total fruit mass, with the skin often being discarded as refuse [19]. As a natural biomass waste composed of cellulose with a porous structure, the durian peel has the advantage of an abundant source, internal special ultra-fine pore structure, and relatively simple constituent (mostly cellulose), which not only can be used as the template for 3D porous skeleton but also the ideal precursor for producing carbon materials [20]. Studies showed that the durian peel contained plentiful alkali metal elements and abundant nitrogen ingredients [21,22]. Urea has found extensive application in agriculture and industry due to its environmentally friendly characteristics, cheap, soluble in water and substantial nitrogen content [23,24]. Considering these advantages, it finds application as a unique N-doped material in heterogeneous materials.

However, few are of interest in the investigation of Fe, N co-doped durian peel materials for oxidation organic pollutions. Hence, the Fe-loading N-doping carbon-based heterogeneous materials based on durian peel, urea, and ferric nitrate were obtained. The structures of obtained materials were measured via scanning electron microscope (SEM), transmission electron microscope (TEM), X-ray diffractometer (XRD), X-ray photoelectron spectroscopy (XPS), and Raman spectra, and the performances of them to activate PMS for oxidation TEC were measured. Radical quenching and electron paramagnetic resonance (EPR) analysis were used to investigate the PMS activation mechanism. This work offered a new method for the comprehensive utilization of durian peel and degradation TEC.

## 2. Results and Discussion

### 2.1. Characterization

**Figure 1:**
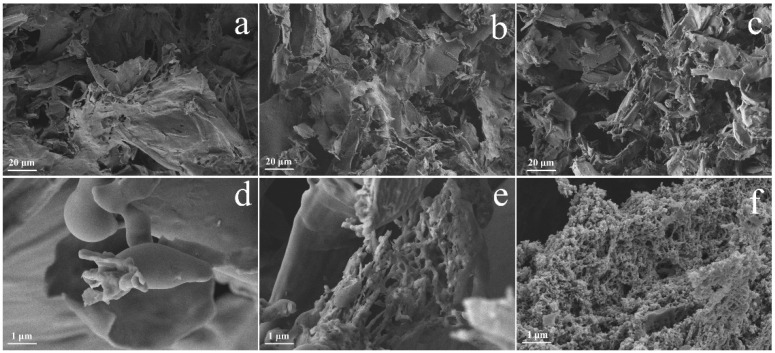
SEM image of EC-700 (**a**,**d**); EC@N_6_-700 (**b**,**e**); EC@N_6_Fe_0.6_-700 (**c**,**f**).

SEM images of various catalyst samples are described in Figure 1. As observed in Figure 1a,d, the pure EC-700 demonstrates a comparatively flat and smooth continuous lamellar structure, accompanied by the presence of holes and folds. Upon the incorporation of urea, the lamellar structure of EC@N_6_-700 underwent a significant refinement, as evidenced in Figure 1b, resulting in the formation of a complex network-like porous structure (Figure 1e). This result is probably due to the existence of an abundant microporous structure in durian peel, which serves as an inducer effectively adsorbing urea molecules within the durian peel tissues. Subsequent decomposition of these adsorbed urea molecules at elevated temperatures leads to the formation of a substantial number of volatile gases, thereby enhancing the porosity of the EC@N_6_-700. In the case of EC@N_6_Fe_0.6_-700, the lamellar structure was found to undergo fragmentation upon the simultaneous doping of urea and iron nitrate (Figure 1d). However, Figure 1f clearly demonstrates that EC@N_6_Fe_0.6_-700 forms a three-dimensional porous mesh structure with many nanoscale particles attached to its surface. This phenomenon can be ascribed to the synergistic effect of abundant urea molecules adsorbed in the durian skin tissue’s internal pore structure and iron nitrate at high temperatures. The process is initiated by the production of volatile gases from the reaction of urea molecules and ferric nitrate at elevated temperatures, leading to the expansion of durian rind pores from within [25]. After this, the reaction of iron ions with surrounding carbon or nitrogen atoms at high temperatures leads to the formation of a significant number of carbon-coated iron nanoparticle structures, thereby further expanding the pores.

**Figure 2:**
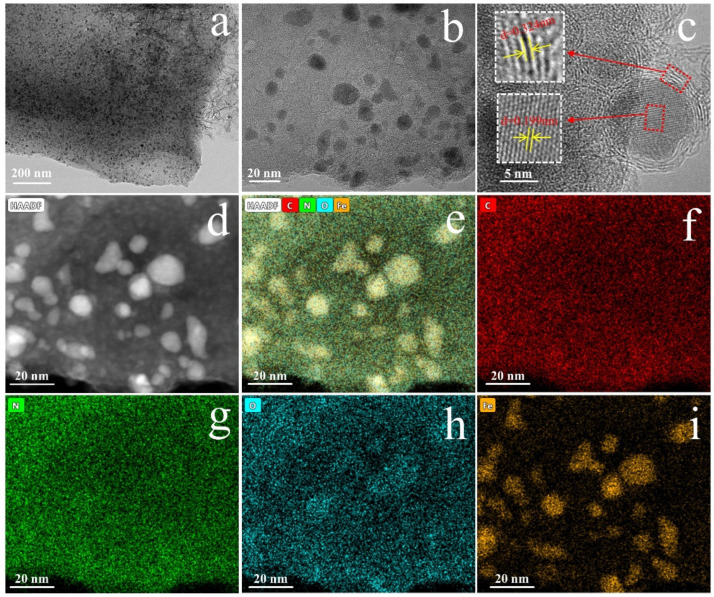
TEM (**a**,**b**); HRTEM (**c**); HAADF-STEM (**d**,**e**); elemental mapping (**f**–**i**).

As demonstrated in Figure 2, the TEM diagram of the EC@N_6_Fe_0.6_-700 discloses the encapsulation of numerous nanoparticles within the carbon layer, with sizes ranging from 5 to 20 nm. Figure 2c illustrates that the spherical nanoparticles are enveloped by a thin, ordered carbon layer, with the calculated values for the lattice stripe spacing of the ordered carbon layer and the spherical nanoparticles being 0.324 nm and 0.199 nm, respectively. These values are assigned to the (002) crystallographic plane of graphitic carbon and (001) crystallographic plane of Fe^0^, suggesting that the EC@N_6_Fe_0.6_-700 is rich in Fe^0^ species [26,27]. This core–shell structure facilitates contact between metal active sites and PMS and pollutants while also effectively preventing the erosion of iron nanoparticles in an acidic environment. The HADDF of EC@N_6_Fe_0.6_-700 and its elemental mapping results are displayed in Figure 2d–i. It is evident that the N element is well-doped on this carbon skeleton, while the Fe element is encapsulated on the N-doped carbon skeleton in dispersed and aggregated forms. This finding suggests that durian peels can be modified by doping to derive Fe-N co-doped carbon-based composites with rich core–shell structures.

**Figure 3:**
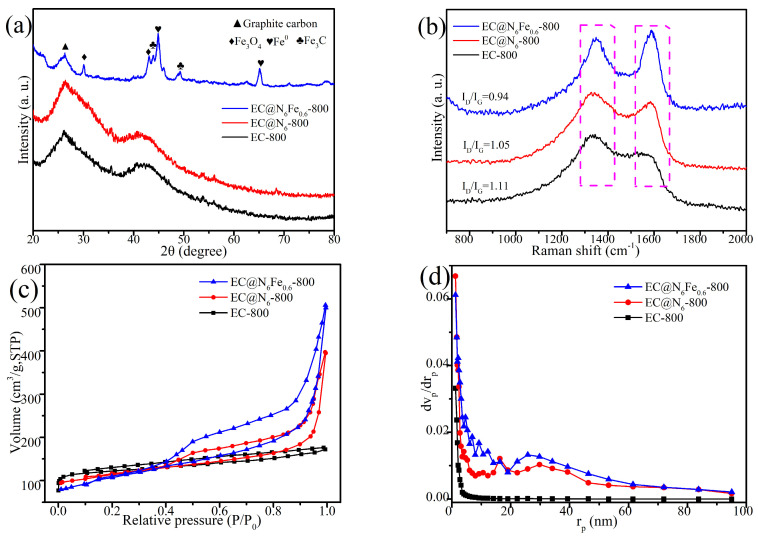
XRD (**a**), Raman (**b**), N_2_ adsorption–desorption isotherms (**c**), pore size (**d**).

The crystal structures of various catalysts were measured by XRD analysis, as illustrated in Figure 3a. A distinct diffraction peak was identified at 26.4° for both EC-700 and EC@N_6_-700 samples, assigned to the (002) crystal plane of graphitic carbon [28]. This observation is attributed to the abundant cellulose content present in durian rind, which serves as an exceptional precursor for the formation of graphitic carbon. Furthermore, the peak intensity of graphitic carbon in EC@N_6_-700 was significantly higher than that of EC-700, which was attributed to the promotion of amorphous carbon ablation by urea under high-temperature conditions, thereby increasing the proportion of graphitic carbon. The peaks of EC@N_6_Fe_0.6_-700 underwent substantial modification following the introduction of iron nitrate. Initially, the intensity of the peak corresponding to graphitic carbon underwent a substantial reduction, attributable to the carbonization and condensation of Fe with graphitic carbon. Secondly, a substantial number of peaks corresponding to Fe were detected. Among them, peaks located at 30°, 43.1°, 43.9°, 49.2°, 44.8°, and 65.1° were assigned to the (220) and (400) crystal planes of Fe_3_O_4_, the (102) and (221) crystal planes of Fe_3_C, and (110) and (200) crystal planes of Fe^0^ (JCPDS No. 19-0629, 35-0772, 06-0696) [29]. This indicates that Fe is present in EC@N_6_Fe_0.6_-700 primarily in the form of Fe^0^.

The Raman spectra of the three materials obtained are described in Figure 3b, which indicates the degree of graphitization in these catalysts. A thorough examination of Raman spectra revealed two prominent peaks at approximately 1350 and 1590 cm^−1^, which are assigned to the D band and G band of carbon. The D-band is attributed to disordered carbon, while the G-band is ascribed to the stretching of carbon atoms with sp^2^ hybridization. Consequently, the I_D_/I_G_ ratio was determined to be a reliable indicator of the degree of graphitization of carbon materials [30]. As illustrated in Figure 3b, the I_D_/I_G_ values of EC-700 and EC@N_6_-700 were 1.11 and 1.05, respectively. This value of EC@N_6_Fe_0.6_-700 was found to be lower than that of EC-700 and EC@N_6_-700, suggesting that the incorporation of metal elements enhances the graphitization of carbon materials.

In Figure 2c, the isotherm of EC-700 is closer to type I, and it produces only a slight hysteresis loop as P/P^0^ increases, which suggests that it has abundant microporous rather than mesoporous structures. However, both EC@N_6_-700 and EC@N_6_Fe_0.6_-700 exhibit typical type IV isotherms, and their adsorption curves show obvious hysteresis phenomena between P/P^0^ values of 0.4~1.0, which suggests that the doped-modified carbon materials are rich in mesoporous structures, and the higher relative pressure, the larger adsorption capacity. The specific surface area (S_BET_) of EC-700, EC@N_6_-700, and EC@N_6_Fe_0.6_-700 is 65.88, 188.65, and 269.35 m^2^ g^−1^, respectively. This shows that doping of moderate urea and Fe elements greatly increased the porosity of the carbon materials. Furthermore, Figure 3d and Table S1 demonstrate that EC@N_6_Fe_0.6_-700 exhibits a higher pore volume (0.64 cm^3^ g^−1^) and pore diameter (21.22 nm) compared to EC-700 (0.07 cm^3^ g^−1^, 5.88 nm) and EC@N_6_-700 (0.44 cm^3^ g^−1^, 17.23 nm). During pyrolysis, the Fe atoms reacted with the surrounding carbon, resulting in an etching effect [31]. Simultaneously, urea generated volatile gases. This combined action resulted in enlarged pore sizes for EC@N_6_Fe_0.6_-700, indicating its substantial S_BET_ and well-developed mesoporous structure. Such characteristics facilitate adequate contact between PMS and contaminants [32].

### 2.2. Optimization of Catalyst

**Figure 4:**
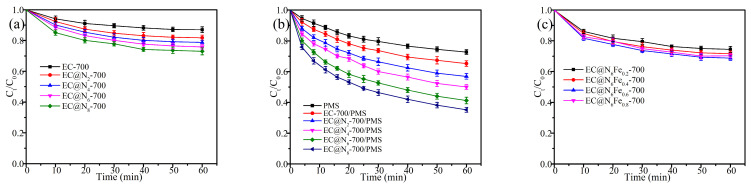

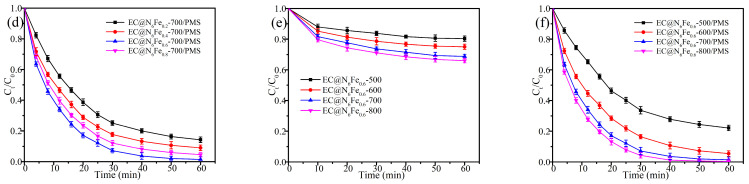
(**a**) urea content on adsorption of TEC, (**b**) urea content on degradation of TEC, (**c**) Fe content on adsorption of TEC, (**d**) Fe content on degradation of TEC, (**e**) carbonization temperature on adsorption of TEC, (**f**) carbonization temperature on degradation of TEC, Conditions: [TEC]_0_ = 20 mg L^−1^, [catalyst]_0_ = 0.10 g L^−1^, [PMS]_0_ = 0.15 g L^−1^, temperature = 25 °C.

As demonstrated in Figure 4, the impact of urea doping, iron nitrate doping, and carbonation temperature on the adsorption and degradation of the catalyst is evident. Figure 4a,b illustrate that the adsorption and catalytic capacity of the catalysts were progressively enhanced with rising of urea doping. This result is because an increased urea content not only facilitates the formation of N-containing active sites at elevated temperatures but also leads to the release of more volatile gases and the enhancement of the catalyst’s pore structure. The adsorption percentage, degradation percentage, and apparent rate constant (k_obs_) of EC@N_2_-700 and EC@N_8_-700 were found to be 24.13%, 58.79%, 0.020 min^−1^ and 26.99%, 64.78%, 0.023 min^−1^ (Figure S1a), respectively, as the ratio of durian peel to urea doping was 1:6 and 1:8, respectively. Despite the higher adsorption and catalytic ability of EC@N_8_-700, considering its lower yield as well as higher production cost, the EC@N_6_-700 was chosen for subsequent experiments.

As demonstrated in Figure 4d and Figure S1b, the introduction of iron nitrate led to a substantial enhancement in the degradation capability of the material. With the rise in iron nitrate doping, the degradation effect of the catalyst on TEC showed an initial rise followed by a subsequent decline. Notably, the EC@N_6_Fe_0.6_-700 demonstrated the optimal degradation capability (98.55%, 0.078 min^−1^) when the iron nitrate to durian peel ratio was 1:0.6. This phenomenon can be ascribed to an appropriate rise in the content of iron nitrate increased the number of Fe-active sites [33]. However, when the content of iron nitrate is excessively high, a large number of iron ions undergo significant agglomeration, which not only blocks some of the pore channels but also reduces the number of nano-Fe active sites [34]. Consequently, the doping amount of iron nitrate was selected as 1:0.6.

As demonstrated in Figure 4e,f, the adsorption and degradation capacity of the catalyst increased with increasing carbonization temperature. The adsorption and degradation capacities and kobs-values (Figure S1c) of EC@N_6_Fe_0.6_-500 and EC@N_6_Fe_0.6_-800 for TEC were 19.68%, 77.88%, 0.030 min^−1^ and 33.99%, 99.58%, 0.102 min^−1^, respectively, as the carbonation temperature increased from 500 °C to 800 °C. This phenomenon was due to the elevated temperatures not only promoted the formation of active sites comprising iron, nitrogen, and carbon, but also facilitated the ablation of amorphous carbon. This, in turn, resulted in an augmentation of the S_BET_ of the catalyst and an escalation in the exposure of active sites. However, adsorption and degradation efficiencies of EC@N_6_Fe_0.6_-700 and EC@N_6_Fe_0.6_-800 were found to be largely comparable, thus necessitating the selection of the carbonization temperature of 700 °C as a means of energy conservation.

Furthermore, to perform a more comprehensive evaluation of the catalytic activity of EC@N_6_Fe_0.6_-700, a comparative analysis was conducted between the performance of EC@N_6_Fe_0.6_-700 activated PMS for the degradation of TEC and that documented in previous reports. The corresponding data are presented in Table S2, which suggests that despite the low concentration of both catalyst and PMS in the EC@N_6_Fe_0.6_-700/PMS/TEC system, its degradation percentage of TEC remains high, suggesting the remarkable performance of the EC@N_6_Fe_0.6_-700/PMS system in removing antibiotics from water. This phenomenon can be attributed to the well-developed pore structure of EC@N_6_Fe_0.6_-700, which ensures effective contact between PMS and TEC with EC@N_6_Fe_0.6_-700. Additionally, the abundant active sites of N-doped carbon-coated iron nanoparticles ensure efficient activation of PMS while avoiding excessive iron leaching.

### 2.3. Evaluation of Catalyst

**Figure 5:**
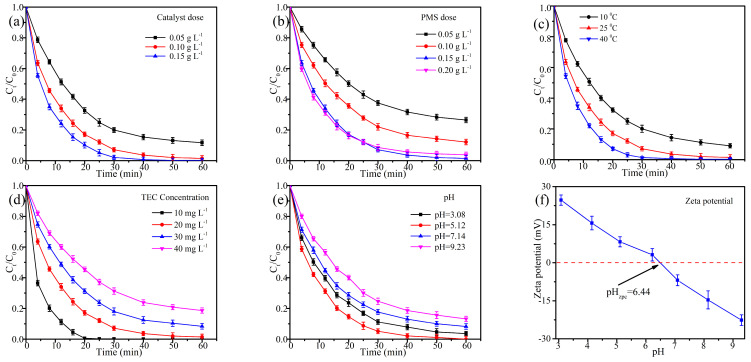
Dose of catalyst (**a**). PMS (**b**). Temperature (**c**). TEC concentration (**d**). pH (**e**). Zeta potential (**f**). Conditions: [TEC]_0_ = 20 mg L^−1^, [EC@N_6_Fe_0.6_-700]_0_ = 0.10 g L^−1^, [PMS]_0_ = 0.15 g L^−1^, temperature = 25 °C.

Considering the intricate and dynamic nature of real water environments, further investigation was conducted into the factors influencing the oxidation efficiency of TEC in EC@N_6_Fe_0.6_-700/PMS systems. As illustrated in Figure 5a and Figure S2a, increasing the catalyst dosage enhanced the removal efficacy. This improvement occurred because a higher amount of EC@N_6_Fe_0.6_-700 provided additional reactive sites and facilitated the production of reactive oxygen species (ROSs) [33]. Specifically, TEC degradation and k_obs_ reached 98.55% and 0.078 min^−1^ at a catalyst concentration of 0.1 g L^−1^. Nevertheless, it is evident that further raising EC@N_6_Fe_0.6_-700 to 0.15 g L^−1^ did not significantly enhance TEC degradation. This suggests that under a fixed PMS dosage, the excess EC@N_6_Fe_0.6_-700 active sites were not fully utilized. Hence, the optimal catalyst dose is 0.10 g L^−1^.

The oxidation efficiency and k_obs_ of TEC under different PMS concentrations were determined and described in Figure 5b and Figure S2b. The production of ROSs, necessary for the oxidation of TEC, occurred through cleavage of O-O bonds in PMS. Hence, the degradation performance of TEC was enhanced from 73.55%, 0.026 min^−1^ to 98.55%, 0.078 min^−1^, with an increase in PMS from 0.05 g L^−1^ to 0.15 g L^−1^. Nevertheless, as PMS reached 0.2 g L^−1^, the oxidation percentage and k_obs_ declined to 96.14% and 0.063 min^−1^. This reduction is probably due to the self-quenching reactions of ROSs [35]. Additionally, the higher k_obs_ between PMS and •OH results in excess PMS being consumed by •OH, thereby reducing its effective utilization.

As shown in Figure 5c and Figure S2c, the impact of reaction temperature on TEC oxidation was determined. It is evident that temperature plays an important role in TEC degradation, with both degradation efficiency and k_obs_ enhancing as temperature rises from 10 °C to 40 °C. This trend indicates an endothermic degradation mechanism. Firstly, higher temperatures enhance molecular thermal motion and reduce diffusion resistance, improving the adsorption of PMS and TEC onto the catalyst surface. Secondly, heating effectively activates PMS, and a higher temperature promotes the formation of ROSs from PMS. At temperatures as low as 10 °C, the degradation performance of TEC still reached 90.99% and 0.045 min^−1^. This further highlights the outstanding PMS activation performance of EC@N_6_Fe_0.6_-700.

Figure 5d and Figure S2d illustrated the oxidation efficiency of TEC at different concentrations under EC@N_6_Fe_0.6_-700/PMS system. The findings suggested that removal efficiency was incrementally decreased as TEC concentration raised from 10 to 40 mg L^−1^. Even as the initial concentration was 40 mg L^−1^, this system also efficiently oxidized over 80% TEC. These results highlight the practical significance of the EC@N_6_Fe_0.6_-700/PMS system for rapid and effective treatment of TEC-contaminated wastewater.

In Figure 5e, the initial pH levels exert a significant influence on the oxidation reaction of TEC. Under varying pH conditions, different functional groups exhibit distinct behaviors, which leads to different charged states of TEC. As the solution’s pH rose from 3.08 to 9.23, the oxidation percentage of TEC initially rose before eventually decreasing. Specifically, at pH levels of 3.08 and 9.23, the oxidation percentage was 96.35% and 86.79%, with corresponding k_obs_ values of 0.060 min^−1^ and 0.041 min^−1^. It suggested that the synthesized EC@N_6_Fe_0.6_-700 maintained impressive catalytic stability across a broad pH spectrum. The potential of EC@N_6_Fe_0.6_-700 was assessed using Zeta potential at various pH levels, leading to the conclusion that its isoelectric point is approximately 6.44. Moreover, it was reported that TEC existed in different forms under various pH. When the pH < 3.2, TEC existed as a cation form (TEC^+^), and the form of it was zwitterion (TEC^0^) under the pH between 3.2 and 8.94, while it existed as an anion form (TEC^−^ or TEC^2−^) under the pH over 8.94 [36,37]. At the pH of 3.08, TEC presents in its TEC^+^ form, while the catalyst exhibits a positive potential, leading to mutual repulsion between TEC^+^ and the catalyst’s surface. This repulsion makes it difficult for TEC to adsorb on EC@N_6_Fe_0.6_-700, thereby impacting the effectiveness of the reaction. At a pH of 9.23, electrostatic repulsion rendered adsorption of TEC unfavorable, while the Fe-active sites on the EC@N_6_Fe_0.6_-700 surface were passivated within the alkaline solution. This resulted in the deposition of Fe^3+^ and hydroxide adsorption, thereby hindering the contact between the active site and PMS. Consequently, the oxidation effect of TEC was inhibited. At a pH of 5, both the negatively charged PMS and the neutral form of TEC can be effectively adsorbed on the EC@N_6_Fe_0.6_-700 surface. This process accelerates the transfer of electrons and the formation of ROSs. Consequently, the degradation efficiency is at its highest at this point.

### 2.4. Influence of Coexisting Ions and Humic Acid

**Figure 6:**
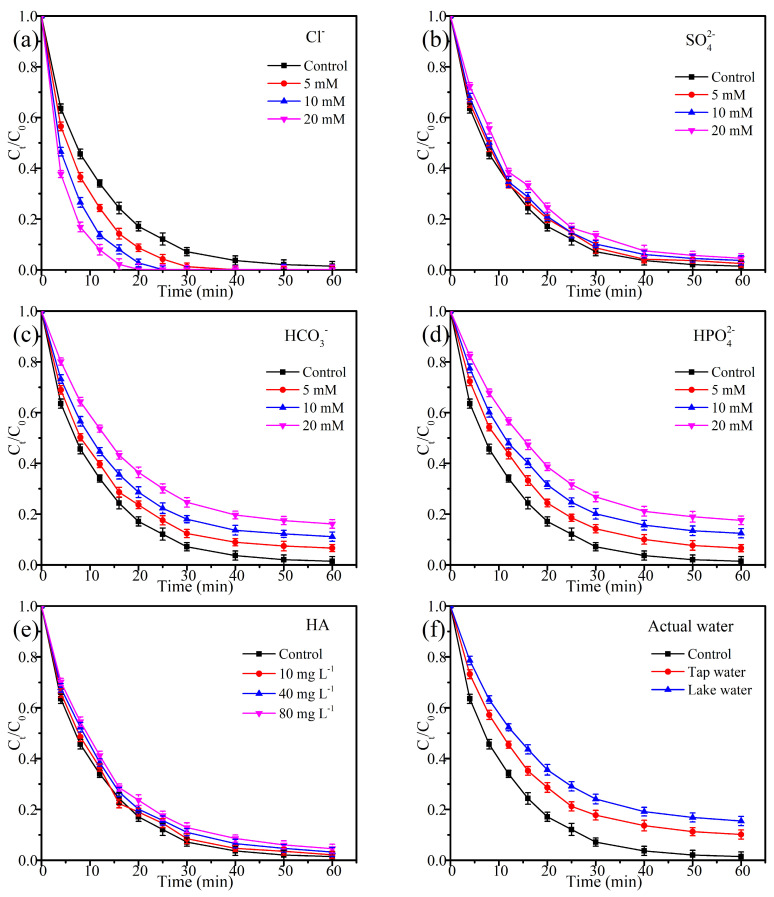
(**a**) CL^−1^, (**b**) SO_4_^2−^, (**c**) HCO_3_^−^, (**d**) HPO_4_^2−^, (**e**) HA, and (**f**) actual water. Conditions: [TEC]_0_ = 20 mg L^−1^, [EC@N_6_Fe_0.6_-700]_0_ = 0.10 g L^−1^, [PMS]_0_ = 0.15 g L^−1^, temperature = 25 °C.

To evaluate the anti-interference capability of EC@N_6_Fe_0.6_-700, the impact of co-existing substances (SO_4_^2−^, HCO_3_^−^, HPO_4_^2−^, Cl^−^, and humic acid) on the oxidation of TEC was measured. As shown in Figure 6a, the existence of Cl^−^ improved the degradation of TEC, with a more significant effect observed at higher Cl^−^ concentrations. The k_obs_ rose from 0.078 to 0.232 min^−1^ as the Cl^−^ concentration increased from 0 to 20 mM (Figure S2a). This enhancement is probably attributed to PMS oxidizing Cl^−^ to generate chlorine radicals and free chlorine species, which in turn expedited the oxidation reaction [38,39]. The existence of SO_4_^2−^ exerts a negligible impact on the TEC oxidation process (Figure 6b and Figure S2b), which is attributed to the low reactivity of SO_4_^2−^ with ROSs.

The existence of HCO_3_^−^ and HPO_4_^2−^ notably hindered the oxidation of TEC (Figure 6c,d). When 20 mM of HCO_3_^−^ and HPO_4_^2−^ were added, the final degradation percentages of TEC decreased to 83.88% and 82.45%, respectively. This reduction was accompanied by a corresponding decrease in k_obs_, which fell to 0.042 and 0.04 min^−1^, respectively. The declined impact can be attributed to multiple factors. First, the pH of the reaction solution increased to 9.01 and 9.77 under the existence of 20 mM HCO_3_^−^ and HPO_4_^2−^, which modified the surface charge of EC@N_6_Fe_0.6_-700 and consequently diminished its adsorption efficiency for TEC [27]. Second, HPO_4_^2−^ can deactivate SO_4_^•−^ and •OH, while ROSs can oxidize HCO_3_^−^, leading to a reduced formation potential of CO_3_^•−^ [40]. Finally, both HCO_3_^−^ and HPO_4_^2−^ can adsorb on the surface of EC@N_6_Fe_0.6_-700, thereby limiting the access of TEC to active sites.

As demonstrated in Figure 6e, the impact of HA on the degradation of TEC is found to be minimal. When HA concentration reached 80 mg L^−1^, the oxidation percentage and k_obs_ of TEC were found to be 90.92% and 0.059 min^−1^, respectively. This assessment reveals that the EC@N_6_Fe_0.6_-700/PMS system exhibits robust performance even in the presence of common interfering compounds, particularly benefiting from the presence of chloride ions. To further evaluate the efficacy of EC@N_6_Fe_0.6_-700 in degrading TEC within actual aquatic environments, experiments were conducted utilizing both lake water and tap water. The final oxidation percentage of TEC in them reduced markedly to 84.55% and 89.88% (Figure 6f). Meanwhile, the corresponding k_obs_ values also declined to 0.044 and 0.053 min^−1^. This indicates that the EC@N_6_Fe_0.6_-700/PMS system maintains effective activity for oxidizing contaminants in real water conditions. To clarify the conditions, an analysis was performed on the parameters of both tap water and lake water. Their hardness levels were measured at 155 and 163 mg L^−1^ (Table 1), suggesting significant concentrations of HCO_3_^−^ and CO_3_^2−^ in both water sources. This is the primary factor responsible for the inhibition of TEC degradation.

### 2.5. Universality and Stability

**Figure 7:**
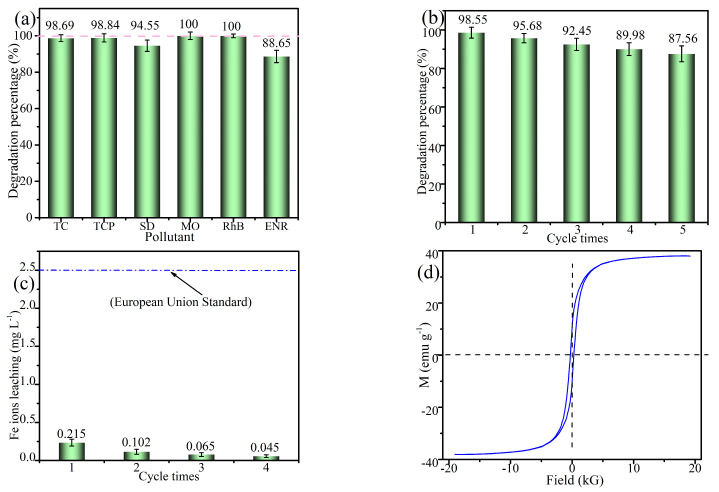
Universality (**a**), reusability (**b**), Fe leaching (**c**), VSM (**d**).

The effectiveness of catalysts in practical applications is significantly impacted by their stability and versatility. To investigate the potential utility of EC@N_6_Fe_0.6_-700, degradation experiments were conducted using a variety of common pollutants. These included tetracycline (TC), trichlorophenol (TCP), sulfadiazine (SD), Enrofloxacin (ENR), rhodamine B (RhB), and methyl orange (MO). As shown in Figure 7a, approximately 98.69% of TC, 98.84% of TCP, 94.55% of SD, 100% of MO, 100% of RhB, and 88.65% of ENR were successfully oxidized. This indicates that EC@N_6_Fe_0.6_-700 exhibits notable versatility in pollutant degradation.

In Figure 7b, the oxidation percentage of TEC declined as the cycle times increased. When the cycle times reached four, the degradation percentage dropped to 87.56%. The reduction can be ascribed to leaching of Fe from EC@N_6_Fe_0.6_-700, oxidation of active sites, and adsorption of oxidation products onto EC@N_6_Fe_0.6_-700. To examine the structural stability of EC@N_6_Fe_0.6_-700, the release of Fe ions in this reaction was evaluated. In Figure 7c, as the cycle times increased from one to four, the leached Fe concentration declined markedly from 0.215 to 0.045 mg L^−1^, falling below the European standards for acceptable limits. Furthermore, these measurements were lower than those reported by other researchers, which is beneficial for the reusability of the catalyst. To evaluate the magnetic properties of EC@N_6_Fe_0.6_-700, its magnetization curve was analyzed. Figure 7d showed that EC@N_6_Fe_0.6_-700 exhibited a significant saturation magnetization value of 39.23 emu g^−1^, demonstrating that it can be efficiently separated from water using an external magnetic field.

### 2.6. Potential Active Site

**Figure 8:**
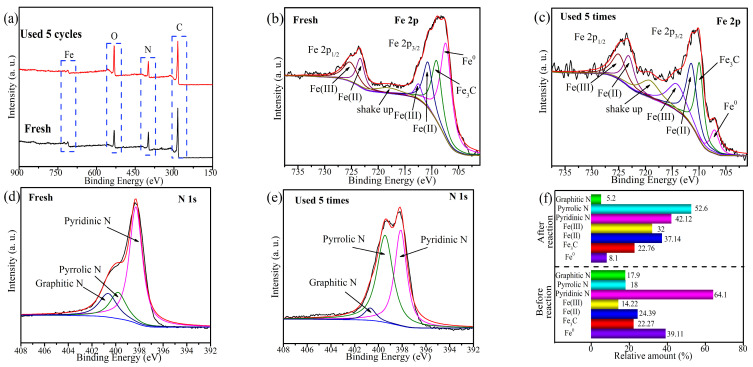
(**a**) XPS, (**b**) fresh of Fe 2p spectrum, (**c**) used of Fe 2p spectrum, (**d**) fresh of N 1s spectrum, (**e**) used of N 1s spectrum, (**f**) relative content.

XPS analysis was employed to analyze the functional groups and surface chemistry of EC@N_6_Fe_0.6_-700 before and after the reaction. Figure 8a shows that the elements of C, N, O, and Fe were detected both prior to and following the reaction. The relative proportions of C, N, and Fe decreased from 67.56%, 17.78%, and 1.65% to 63.97%, 14.96%, and 1.11% as the cycle times increased to five. Concurrently, the proportion of O rose from 13.01% to 19.96%. This phenomenon can be explained by the redox reactions and the accumulation of intermediates on the EC@N_6_Fe_0.6_-700 surface that occur during the catalytic degradation process. In Figure 8b,c, the Fe 2p spectrum of EC@N_6_Fe_0.6_-700 can be decomposed into seven distinct peaks, corresponding to Fe^0^ (707.3 eV), Fe_3_C (709.1 eV), Fe(II) 2p3/2 (710.7 eV), Fe(III) 2p3/2 (712.5 eV), Fe(II) 2p1/2 (723.4 eV), Fe(III) 2p1/2 (725.2 eV), and shake-up satellites (718.4 eV) [12,40]. Notably, after five cycles, the proportion of Fe^0^ declined significantly from 39.11% to 8.1%. In contrast, the values of Fe(II) and Fe(III) increased from 24.39% and 14.22% to 37.14% and 32%, respectively (Figure 8f). This suggests that Fe participates in the oxidation cycles, with Fe^0^ likely serving as an active site and undergoing transformations between Fe(II) and Fe(III) states. The N 1s spectrum depicted in Figure 8d,e identified three distinct nitrogen species: pyridine N (398.2 eV), pyrrole N (399.7 eV), and graphite N (400.7 eV) [41]. These N species play an important role in modulating the chemical reactivity of EC@N_6_Fe_0.6_-700. Specifically, pyridine N enhances electron transfer within the sp^2^ carbon network, which assists EC@N_6_Fe_0.6_-700 in activating PMS to generate ROSs. Meanwhile, graphite N serves as an active site for the nonradical pathway, facilitating the production of ^1^O_2_. After five reaction cycles, there was a decline in the content of pyridine N and graphitic N, which decreased from 64.1% and 17.9% to 42.12% and 5.2% (Figure 8f). It demonstrated that pyrrolic N and graphitic N are the essential active sites for PMS activating.

### 2.7. Catalytic Mechanisms

**Figure 9:**
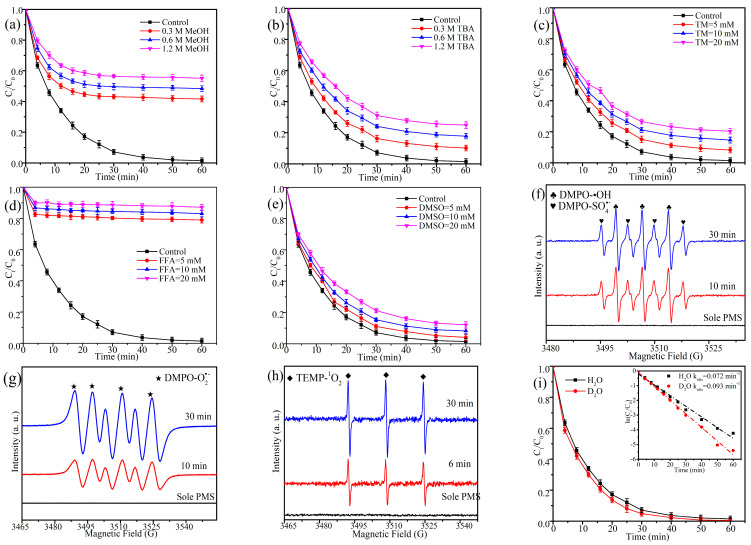

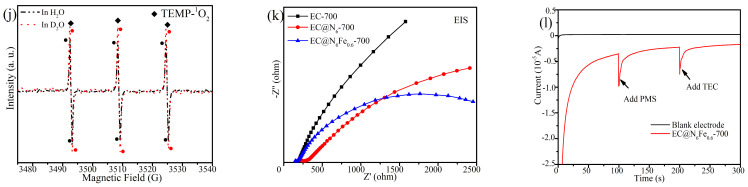
Quenching experiments (**a**–**e**). EPR spectra (**f**–**h**,**j**). D2O exchange experiment (**i**). EIS (**k**). IC (**l**).

To identify the presence of ROSs during the oxidation of TEC, various scavenger was used to quench the TEC oxidation process. The various scavengers exhibited differing impacts on the TEC oxidation reaction. Figure 9a,b demonstrated that around 55.12% and 24.89% inhibition of TEC degradation was produced under 1.2 M of MeOH and TBA. It is important to observe that MeOH can effectively eliminate both SO_4_^•−^ and •OH simultaneously, whereas TBA exhibits a significantly higher affinity for quenching •OH compared to SO_4_^•−^. Consequently, the disparity in degradation efficiencies between MeOH and TBA reflects the contribution of SO_4_^•−^ to the overall process. It indicated that SO_4_^•−^ and •OH play partial roles in TEC degradation. Figure 9c showed that the inhibition percentage of TEC was increased from 8.23% to 20.35% as the existence of trichloromethane (TM) rose from 5 mM to 20 mM, suggesting that O_2_^•−^ was also involved in this reaction. The presence of furfuryl alcohol (FFA) confirmed the existence of ^1^O_2_ (Figure 9d). The inhibition percentage of TEC was significantly increased to 79.01% under the incorporation of 5 mM FFA, and the inhibition impact was increased as FFA concentration increased, suggesting that ^1^O_2_ obviously facilitated TEC oxidation. Moreover, research has demonstrated that high-valent iron-oxo intermediates (Fe^IV^ = O) can potentially form in iron-based materials to activate PMS. To verify this, dimethyl sulfoxide (DMSO) was utilized as a scavenger. The presence of DMSO had minimal impact on the degradation of TEC (Figure 9e), suggesting that the role of Fe^IV^ = O in this system is relatively insignificant.

To mitigate the possible inaccuracies in the quenching outcomes, EPR detection was used to analyze the formed ROSs. In Figure 9f–h, the presence of peaks associated with DMPO-•OH, DMPO-SO_4_^•−^, DMPO-O_2_^•−^, and TEMP-^1^O_2_ was detected. The intensity of these peaks enhanced as the reaction progressed, indicating the production of SO_4_^•−^, •OH, O_2_^•−^, and ^1^O_2_ species within this reaction. Others’ studies have indicated that the inhibition of FFA might result from direct interactions between FFA and PMS rather than the scavenging of ^1^O_2_. Additionally, the TEMP-^1^O_2_ signal probably originated from electron transfer between TEMP and the catalyst/PMS system rather than from the actual production of ^1^O_2_ [41]. Hence, oxidation experiments and EPR detection were performed using D_2_O as a solvent. In Figure 9i, the k_obs_ value and oxidation efficiency of TEC in D_2_O were higher than that in H_2_O. Additionally, the intensity of the TEMP-^1^O_2_ peak in D_2_O was more pronounced compared to that observed in H_2_O under identical conditions. These findings suggest that the generation of ^1^O_2_ was significant and played an important role in TEC degradation.

Considering the inherent trend of electron transfer during the activation of PMS by carbon-based materials, electrochemical impedance spectroscopy (EIS) and instantaneous current (IC) were employed to investigate the electron transfer mechanism on the surface of EC@N_6_Fe_0.6_-700 [42]. In Figure 9k, EC@N_6_Fe_0.6_-700 displayed a reduced semicircle diameter relative to EC@N_6_-700 and EC-700, indicating that Fe-N co-doped modified durian peel exhibited a low charge transfer resistance and efficient electron transfer within the EC@N_6_-700 architecture. The lower charge transfer resistance is not only attributed to the existence and well-distributed Fe nanoparticles but is also associated with the material’s S_BET_ and pore size [43]. IC was conducted to verify the electron transfer from TEC to PMS. Figure 9l described that the introduction of PMS did not induce notable changes at the bare glassy carbon electrode. In contrast, a substantial decrease in current was detected at the working electrode modified with EC@N_6_Fe_0.6_-700. Research indicated that incorporating nitrogen into carbon-based catalysts enhances electron exchange between the catalyst and PMS, resulting in a metastable oxidized state [32]. When TEC was added, the working electrode containing EC@N_6_Fe_0.6_-700 exhibited a significant change in current, demonstrating the catalyst’s ability to effectively manage electron transfer from the pollutant to PMS.

Hence, the possible mechanism for the oxidation of TEC using EC@N_6_Fe_0.6_-700 activated PMS is illustrated. Initially, EC@N_6_Fe_0.6_-700 acts as an electron mediator by offering adsorption sites for TEC (acting as an electron donor) and catalytic active sites for PMS activation (serving as an electron acceptor) [32]. The TEC molecules in the solution adhere to the EC@N_6_Fe_0.6_-700 surface via strong π-π interactions. Upon introducing PMS, the graphitic N on the EC@N_6_Fe_0.6_-700 surface facilitates rapid electron transfer between PMS and TEC, enabling swift electron donation from TEC to PMS [44]. Simultaneously, upon accepting electrons, the O-O bond of PMS cleaves to form ^1^O_2_. This process of electron transfer and ROS production leads to the breakdown of TEC into smaller molecules, ultimately forming non-toxic CO_2_ and H_2_O.

### 2.8. Degradation Pathways of TEC and Toxicological Assessment

**Figure 10:**
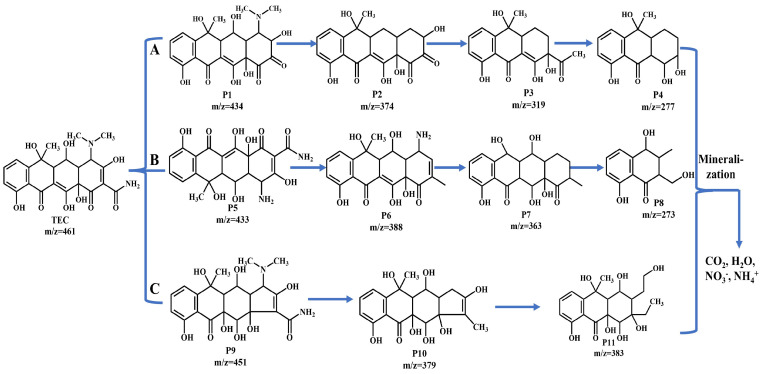
Degradation pathways of TEC (path A, B, and C).

HPLC-MS successfully identified various degradation products generated following TEC decomposition (Table S3), allowing for the deduction of potential degradation pathways that occur during this process (Figure 10). Pathway A involves a sequential process of bond cleavage in TEC. The formation of P1 (*m*/*z* = 434) results from the diacylamino reaction of TEC. Subsequently, the removal of the dimethylamino group from P1 leads to the creation of P2 (*m*/*z* = 374). P2 then undergoes hexatomic ring cleavage to produce P3 (*m*/*z* = 319). P3 continues to break the branches of the six-membered ring, forming P4 (*m*/*z* = 277) [37]. In Pathway B, TEC undergoes further transformation to generate P5 (*m*/*z* = 433) through the cleavage of C = C and N-C bonds. Subsequently, the cleavage of the C = O and N-C bonds in branches leads to the generation of P6 (*m*/*z* = 388). Then, the removal of amino and methyl groups and the cleavage of the C = C bond of P6 to produce P7 (*m*/*z* = 363). Finally, the hexatomic ring cleavage of P7 leads to the generation of P8 (*m*/*z* = 273). In pathway C, P9 (*m*/*z* = 451) was generated by the elimination of carbonyl groups. Then, the diacylamino reaction and removal dimethylamino group of P9 resulted in the generation of P10 (*m*/*z* = 379). The cleavage of the C = C bond on the penta ring of P10 results in P11 (*m*/*z* = 383). Ultimately, the TEC undergoes mineralization, breaking down into smaller molecules that are eventually converted into H_2_O, NO_3_^−^, NH_4_^+^, and CO_2_.

The toxicity of intermediates serves as a crucial factor of the oxidation system’s advantages and limitations, requiring thorough assessment. The aquatic toxicity of the intermediates was comprehensively evaluated using the Ecological Structure–Activity Relationship (ECOSAR) model [45]. The acute (LC50 or EC50) and chronic (ChV) toxicity values were measured according to the standard (Table S4) [46]. The LC50 values for TEC were calculated to be 80,200 mg/L for fish and 5660 mg/L for daphnia, while the EC50 value for green algae was 1320 mg/L (Table 2). Additionally, the chronic toxicity levels of TEC for fish, daphnia, and green algae were all harmless. Following the process of degradation, it was observed that all products exhibited chronic non-toxicity to fish. Concurrently, all products exhibited acute non-toxicity to fish, Daphnia, and green algae. It can be inferred that the EC@N_6_Fe_0.6_-700/PMS system efficiently oxidizes TEC in water without causing ecological biotoxicity to the environment. This confirms its environmental sustainability and suitability for practical applications.

## 3. Materials and Methods

### 3.1. Materials

The information on chemical reagents is shown in Text S1.

### 3.2. Catalyst Preparation

Durian peel was washed with water and cut into pieces, then it was put in an oven and dried at 45 °C. Next, the dry durian peel was pulverized through a sieve of 100 mesh. In the synthesis process, the calculative amount of Fe(NO_3_)_3_·9H_2_O and urea were dissolved in deionized water. Moreover, 2 g of dry durian peel powder was added and stirred for 10 min. Subsequently, the above mixture was dried at 60 °C. After that, the dry solid was pyrolyzed at 700 °C for 3 h under N_2_ protection. The synthetic materials were named DCNxFey-Z (x, y represent mass ratio of urea and Fe(NO_3_)_3_·9H_2_O to durian peel, Z represents the carbonization temperature). In addition, the materials of DC (pure durian peel) and DCNx (without doping Fe(NO_3_)_3_·9H_2_O) were obtained in the same process.

### 3.3. Analytic Methods

The catalytic and analytic methods are shown in Text S2 [47,48,49,50,51,52,53,54].

## 4. Conclusions

In this research, the EC@N_6_Fe_0.6_-700 catalyst was synthesized through a one-step carbonization process, utilizing durian peel loaded with urea and ferric nitrate as the precursor material. The EC@N_6_Fe_0.6_-700 was then employed to activate PMS for oxidation of TEC. The incorporation of an optimal amount of urea and ferric nitrate improved the catalytic activity. The Fe nanoparticles, encapsulated by N-doped graphene shells, offer numerous active sites for PMS activation and significantly decrease the leaching of Fe. ^1^O_2_ is the primary ROS in the EC@N_6_Fe_0.6_-700/PMS system for TEC oxidation. The synthesized EC@N_6_Fe_0.6_-700 material effectively oxides TEC within 60 min and exhibits resistance to interference, reusability, and robust stability throughout the oxidation process. Consequently, this research will contribute to the synthesis of environmentally friendly, heterogeneous materials with enhanced stability, thereby enhancing practical applications in wastewater treatment.

## Figures and Tables

**Table 1:** Index parameters in actual water.

Water	pH	Hardness (CaCO_3_ mg L^−1^)	SO_4_^2−^ (mg L^−1^)	Cl^−^ (mg L^−1^)	DOC (mg L^−1^)
Lake water	7.12	163	41.02	0.09	8.55
Tap water	6.65	155	40.85	0.73	1.46

**Table 2:** Toxicity of TEC and its intermediates as predicted by ECOSAR.

Compound	Acute Toxicity (mg L^−1^)			Chronic Toxicity (mg L^−1^)			Long Kow	Water
	Fish	Daphnid	Green Algae	Fish	Daphnid	Green Algae		Solubility
	(LC_50_, 96 h)	(LC_50_, 48 h)	(EC_50_, 96 h)	(ChV)	(ChV)	(ChV)		(mg L^−1^)
**CIP**	8.02 × 10^4^	5.66 × 10^3^	1.32 × 10^3^	2.30 × 10^4^	280	2.99 × 10^3^	−2.5012	32,596
**P1**	2.44 × 10^4^	1.32 × 10^5^	5.20 × 10^5^	1.58 × 10^6^	5.02 × 10^3^	9.76 × 10^4^	2.2306	158.93
**P2**	2.71 × 10^6^	1.03 × 10^6^	1.43 × 10^5^	1.64 × 10^5^	3.24 × 10^4^	1.52 × 10^4^	−2.3929	1,000,000
**P3**	1.36 × 10^3^	719	395	122	57.1	87.8	1.2015	3430.5
**P4**	5.04 × 10^3^	2.49 × 10^3^	1.05 × 10^3^	419	166	202	0.5043	23,109
**P5**	2.1 × 10^5^	1.37 × 10^4^	3.72 × 10^4^	7.65 × 10^4^	626	7.99 × 10^3^	−3.1783	185,500
**P6**	8.71 × 10^3^	720	1.23 × 10^3^	1.55 × 10^3^	41.4	313	−1.1483	133,220
**P7**	1.66 × 10^4^	7.89 × 10^3^	2.80 × 10^3^	1.31 × 10^3^	467	491	0.0549	17,788
**P8**	1.09 × 10^3^	573	307	97.2	44.7	67.2	1.1349	136,688
**P9**	2.16 × 10^6^	1.18 × 10^5^	4.56 × 10^5^	1.36 × 10^6^	4.53 × 10^3^	8.62 × 10^4^	−4.7168	1,000,000
**P10**	2.25 × 10^4^	1.06 × 10^4^	3.57 × 10^3^	1.76 × 10^3^	606	611	−0.0717	18,220
**P11**	5.16 × 10^3^	2.59 × 10^3^	1.15 × 10^3^	435	178	228	0.6465	4192.3
		Very toxic		Toxic		Harmful		Harmless

Red—very toxic; pink—toxic; yellow—harmful; green—harmless.

## Data Availability

Please contact the corresponding author for specific experimental data.

## Supplementary Materials

The following supporting information can be downloaded at: www.mdpi.com/article/10.3390/molecules30051005/s1. Text S1. Materials; Text S2. Characterization of catalyst and analytic methods; Table S1. BET surface area and pore properties of catalysts; Table S2. Possible intermediates for TEC degradation; Table S3. Toxicity classification according to the Globally Harmonized System of Classification and Labelling of Chemicals; Table S4. Toxicity classification according to the Globally Harmonized System of Classification and Labelling of Chemicals; Figure S1. Effect of various catalysts on kobs of TEC; Figure S2. Catalyst dose (a), PMS dose (b), reaction temperature (c), TEC concentration (d), and pH (e) on the kobs of TEC; Figure S3. Effect of inorganic anions, HA, and actual water on the kobs of TEC. References [47,48,49,50,51,52,53,54] are cited in Supplementary Materials.
