# Supplementary material for: Upcycling of Waste Durian Peel into Valued Fe/N Co-Doped Porous Materials as Peroxymonosulfate Activator for Terramycin Oxidation
Source: Molecules. 2025 Feb 21;30(5):1005. doi: 10.3390/molecules30051005 (PMC11901486; doi:10.3390/molecules30051005)
Supplement: Supplementary file 1 [file molecules-30-01005-s001.zip › molecules-3474174-supplementary.pdf]

Supporting Information

Upcycling of waste durian peel into valued Fe/N co-doped porous materials as peroxymonosulfate activator for terramycin oxidation

Kewang Zheng[a], Rui Liu[a], Lihang Shen[b], Wei Li[a]*, Caiqin Qin[a*]

a. School of Chemistry and Materials Science, Hubei Engineering University, Xiaogan, China

b. Faculty of Engineering and Computing, The University of Sydney, Sydney, Australia

*E-mail: Wei Li (E-mail: weili@hbeu.edu.cn), Qincq (E-mail: qincq@hbeu.edu.cn)

List of Text, Tables and Figures

Text S1. Characterization of catalyst and analytic methods

Table S1 BET surface area and pore properties of catalysts

Table S2 Possible intermediates for TEC degradation.

Table S3. Toxicity classification according to the Globally Harmonized System of Classification and Labelling of Chemicals.

Fig. S1 Effect of various catalysts on k_{obs} of TEC

Fig. S2 catalyst dose (a), PMS dose (b), reaction temperature (c), TEC concentration (d) and pH(e) on the k_{obs} of TEC

Fig. S3 Effect of inorganic anions, HA and actual water on the k_{obs} of TEC

Text S1. Materials

Durian peel was obtained from local supermarket. Urea, ferric chloride, PMS, sodium bicarbonate, sodium chloride, sodium nitrate, sulfuric acid, acetic acid and other reagents were obtained from Shanghai Titan Technology Co., LTD, China. CIP and other contaminants were purchased from Aladdin Industrial Corporation, China. All reagents were of analytical grade and used without further purification.

Text S2. Characterization of catalyst and analytic methods

The microstructure of the prepared catalysts was determined by scanning electron microscopy (SEM, TESCAN CZECH) and transmission electron microscopy (TEM, TF20). High angle annular dark feld scanning TEM (HAADF-STEM) images and corresponding elemental mapping were obtained by FEI FT20 TEM/STEM. The X-ray diffraction (XRD) pattern of catalysts powder was analyzed with an X-ray Diffractometer (Bruker D8 Advanced, Cu Kα radiation, λ = 0.15406 Å, Germany). X-ray photoelectron spectroscopy (XPS) of the catalysts was performed using an ESCALAB 250 XI (Thermo Fisher, US) spectrometer. Raman spectra were collected on a Horiba Scientific LabRAM HR Evolution Raman spectrometer. N_2 adsorption-desorption isotherm and the Brunauer-Emmett-Teller (BET) surface area of the catalysts were evaluated through ASAP 2460. The magnetic properties of the sample were measured by a vibrating sample magnetometer (VSM) and a Quantum Design PPMS-9 physical property measurement system. Electron paramagnetic resonance (EPR) signals were collected on a Bruker EMS-plus instrument with Xeon software (Bruker). Zeta potentials of Fe@CN-8 in solutions of different pH were measured using Zeta potential analyzer (Zetasizer Nano ZSE, Malvern, UK), then calculated the pH_{PZC} of the catalyst.

The degradation experiments of the prepared catalysts for PMS activation were determined by degradation of TEC. Generally, 10 mg of catalysts was added to 100 mL pollutant solution (20 mg/L). Then, PMS (0.15 g/L) was added to the solution, the reaction was carried out on a magnetic stirrer at around 25 °C. At certain time

intervals, aliquots (2.0 mL) were extracted from the solution and filtered with a 0.22 µm PES filter, 0.1 mL of $Na_2S_2O_3$ (0.1 M) was mixed with 1 mL of the filtered solution to terminate the reaction, and the concentration of residual TEC in the filtered solution was analyzed by high performance liquid chromatography (HPLC). To investigate the reusability of the catalyst, the used catalyst was washed with deionized water and ethanol for 2 times, dried at 60 ˚C, and then used for the next cycle.

Removal percentage (%) = $(C_0\text{-}C_t)$ ×100%

$\ln (C_t/C_0) = -k_{obs}\, t$

Where: C_0 and C_t are the liquid phase peak areas (or UV absorbance values) of the pollutants in the solution at the initial and t (min) moments, respectively; Removal percentage (%) is the degradation efficiency of the pollutants, kobs is the apparent rate constant of the reaction (degradation rate), and t is the reaction time.

High performance liquid chromatography (HPLC) analysis conditions: chromatographic column was Thermo C18 column (5 µm, 4.6 mm × 150 mm); injection volume was 10 µL, and the column temperature was 25 °C.

Analysis conditions

Pollutants	Mobile phase	Volume ratio	Flow rate ($mL\ min^{-1}$)	Detection wavelength (nm)
TEC	Acetonitrile/0.1% oxalic acid	30/70	1.0	357
2,4,6-TCP	Methanol/water	80/20	1.0	290
SDZ	Methanol/water	85/15	1.0	270
4-NP	Methanol/ water	70/30	1.0	317

The concentration of Rhodamine B and Orange II were evaluated by an UV-Vis spectrophotometer (Shimazu UV-2700) with incident wavelength of 554 nm and 484 nm, respectively.

The leaching of iron ion from the catalyst at different pH was measured by an 8000DV Inductively Coupled Plasma-Optical Emission Spectrometer (ICP-OES, Perkin Elmer, US).

The concentration of PMS in the reaction solution was determined by the method of UV spectrum. 1 mL of the filtered reaction solution was mixed with 4 mL of KI stock solution (containing 10 mM KI and 5 mM $NaHCO_3$), then the mixture reacted in dark for 5 min; after it, the absorbance value of the mixture solution was determined at 352 nm.

The concentration of H_2O_2 produced in the system was analyzed by the method of N, N-diethyl-p, phenylenediamine (DPD). The experimental procedure was as follows. 1) 50 mg of DPD was dissolved in 5 ml of 0.05 M H_2SO_4, named as solution A. 2) 5 mg of peroxidase (POD) was dissolved in 5 mL of water, named as solution B. 3) 10 mL of 0.1 M Na_2HPO_4 solution was mixed with 90 mL of 0.1 M NaH_2PO_4 solution to obtain solution C. 4) 3 mL of deionized water, 0.3 mL of solution C, 30 μL of solution A and 30 μL of solution B were mixed to obtain solution D. 5) 1 mL of the filtered solution was mixed with solution D, and the absorbance value was measured at 551 nm.

The mechanism of electron transfer of non-free radicals in the system was measured by chronoamperometry analysis. A standard three-electrode electrochemical cell with an Ag/AgCl reference electrode, a platinum wire counter electrode and a catalyst-modified glassy carbon working electrode was used, and the electrolyte was 0.1 M Na_2SO_4. The working electrode was obtained as follows: 5 mg catalyst powder, 0.1 mL Nafion solution (5 wt%, Sigma-Aldrich) and 1 mL absolute N, N-Dimethylformamide were mixed and ultrasound for 5 min. Then, 10 μL of the as-prepared mixture was dripped onto the surface of the polished glassy carbon electrode and dried in ambient air before use. The data was obtained by a CHI 760D electrochemical workstation (Chenhua, China).

Table S1 BET surface area and pore properties of catalysts

Sample	S_{BET} [m^2 g^{-1}]	Vp [cm^3 g^{-1}]	Mean pore diameter (nm)
EC-700	65.88	0.07	5.88
EC@N_6-700	188.65	0.44	17.23
EC@$N_6Fe_{0.6}$-700	**269.35**	**0.64**	**21.22**

Table S2 Comparison with previously reported catalysts

Catalyst	Dosage(g L^{-1})	PMS(g L^{-1})	TEC(mg L^{-1})	Time(min)	Removal(%)	Reference
$KNBC750_4$	0.1	1.35	15	30	100%	**[1]**
Fe_3O_4/Co-Mn_3O_4	0.2	2.70	10	60	96.0%	**[2]**
ZIF-67	0.1	0.54	10	10	87.73%	**[3]**
Co3O4-c	0.5	0.5	20	60	82.8	**[4]**
OVs-$CuFe_2O_4$	20	0.27	20	45	95.7%	**[5]**
MAC	0.1	0.14	20	60	100%	**[6]**
Co_3O_4 – CuO@CN-2	0.05	0.54	20	60	97.1%	**[7]**
Co@C-600	0.1	0.15	10	15	88.5%	**[8]**
**EC@$N_6Fe_{0.6}$-700**	**0.1**	**0.15**	**20**	**60**	**98.55**	**This work**

Table S3 Possible intermediates for CIP degradation.

Products	Molecular formula	m/z	Structural formula
**TEC**	$C_{22}H_{24}N_2O_9$	461	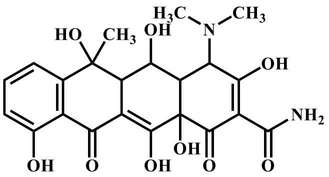
**P1**	$C_{21}H_{23}NO_9$	434	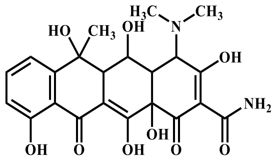
**P2**	$C_{19}H_{18}O_8$	374	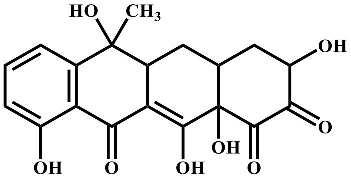
**P3**	$C_{17}H_{18}O_6$	319	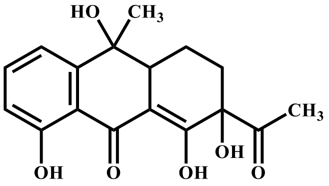
**P4**	$C_{15}H_{16}O_5$	277	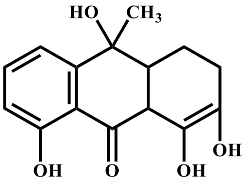
**P5**	$C_{20}H_{20}N_2O_9$	433	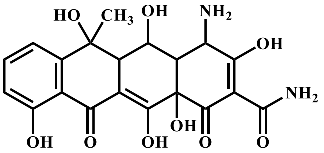
**P6**	$C_{20}H_{21}NO_7$	388	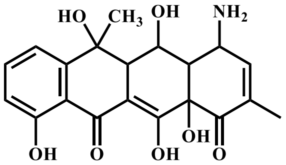
**P7**	$C_{19}H_{22}O_7$	363	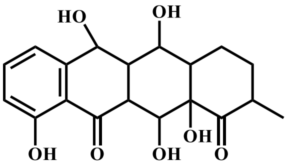

**P8**	$C_{12}H_{14}O_4$	273	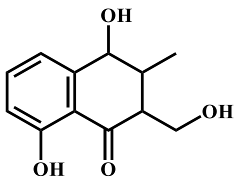
**P9**	$C_{21}H_{26}N_2O_9$	451	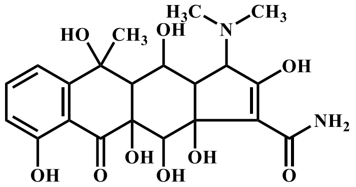
**P10**	$C_{19}H_{22}O_8$	379	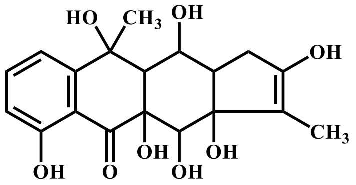
**P11**	$C_{19}H_{26}O_8$	383	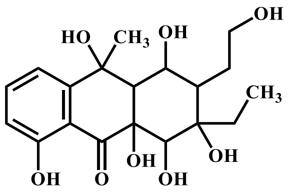

Table S4. Toxicity classification according to the Globally Harmonized System of Classification and Labelling of Chemicals.

**Toxicity range (mg L^{-1})**	**Class**
LC_{50}/ChV ≤1	Very toxic
1< LC_{50} /ChV ≤10	Toxic
10< LC_{50}/ChV ≤100	Harmful
LC_{50}/ ChV >100	Not harmful

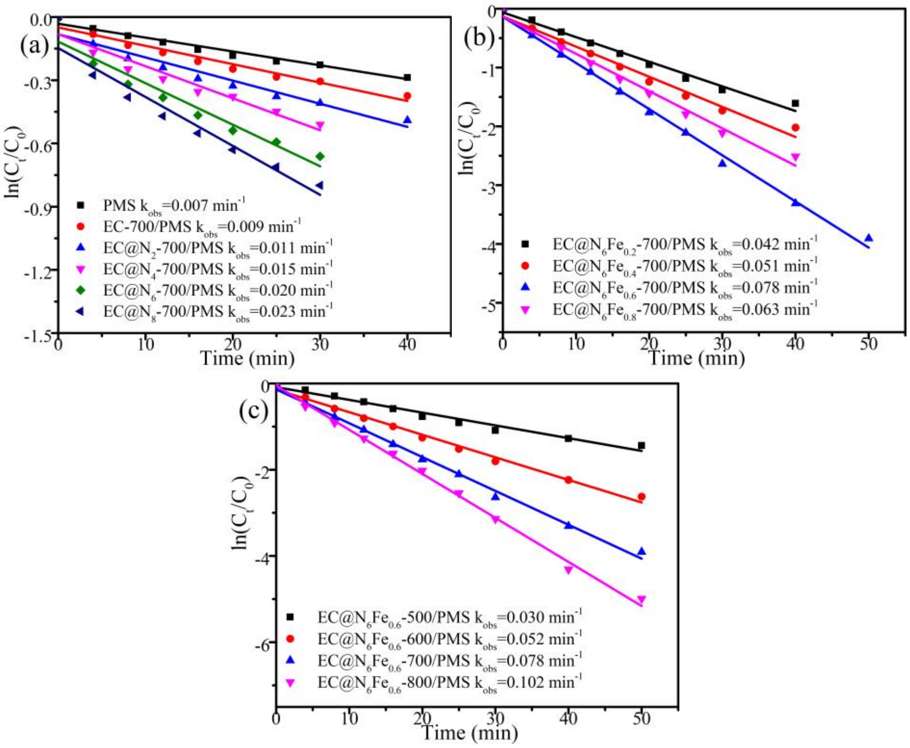

Fig. S1 Effect of various catalysts on k_{obs} of TEC

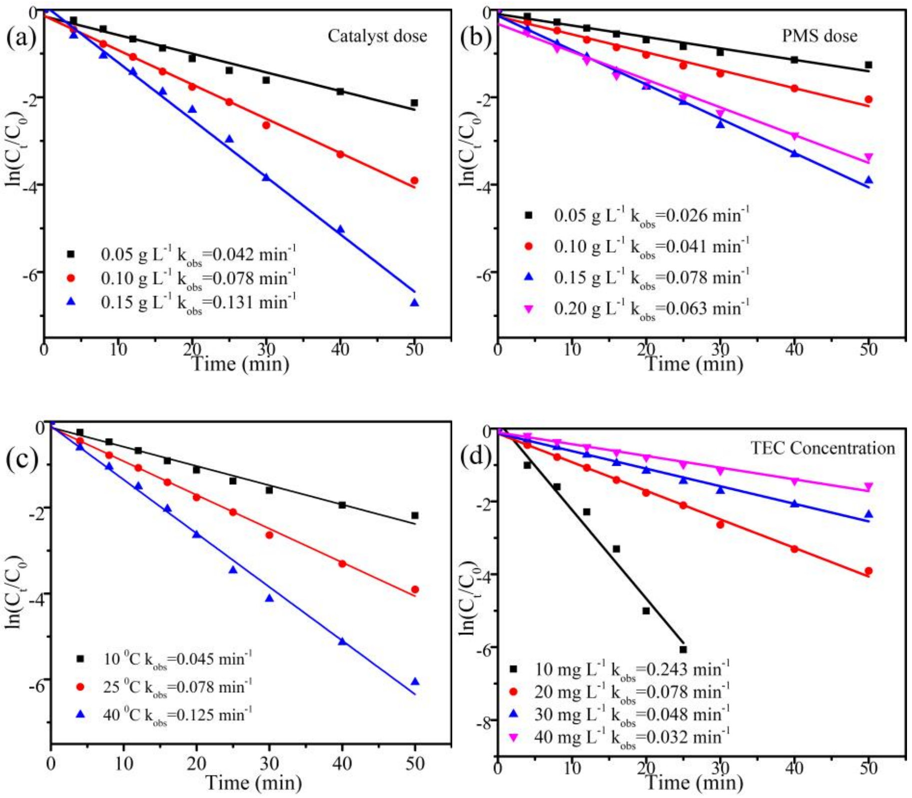

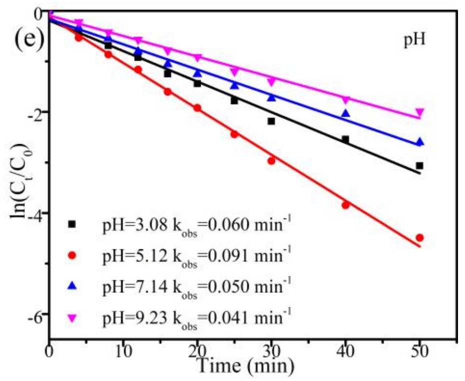

Fig. S2 catalyst dose (a), PMS dose (b), reaction temperature (c), TEC concentration (d), pH (e) on the k_{obs} of TEC

Conditions: $[CIP]_0$ =20 mg L^{-1}, $[catalyst]_0$ =0.15 g L^{-1}, $[PMS]_0$ = 0.2 g L^{-1}, initial pH = 3.89, temperature = 25℃.

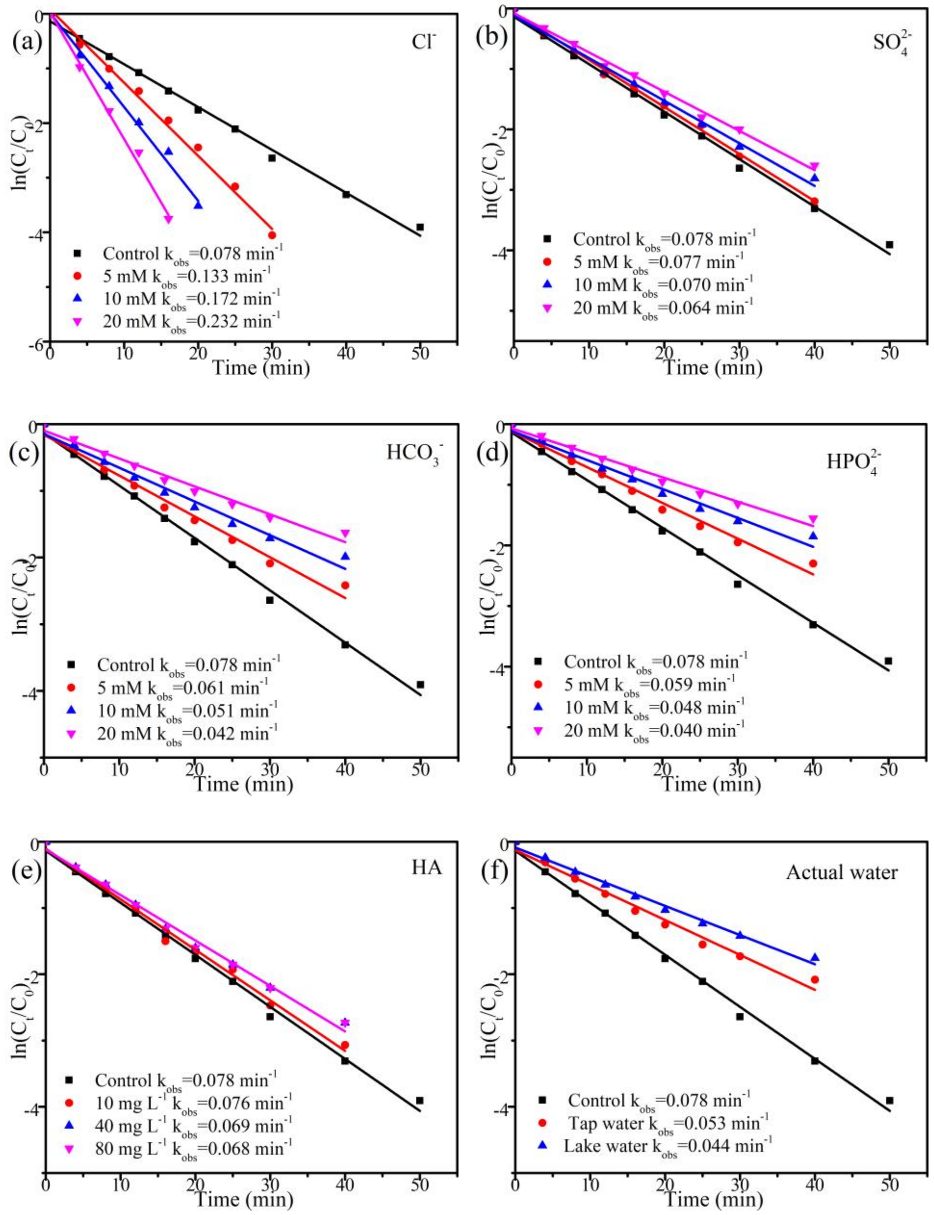

Fig. S3 Effect of inorganic anions, HA and actual water on the k_{obs} of TEC

Conditions: $[CIP]_0$ =20 mg L^{-1}, $[catalyst]_0$ =0.15 g L^{-1}, $[PMS]_0$ = 0.2 g L^{-1}, initial pH = 3.89, temperature = 25℃.

[1] I.R. Akaniro, R. Zhang, X. Chai, C.H. Tsang, P. Wang, S. He, Z. Yang, J. Zhao, Engineered digestate-derived biochar mediated peroxymonosulfate activation for oxytetracycline removal in sustainable wastewater remediation, Environmental Pollution 360 (2024) 124640.

[2] L. He, H. Li, J. Wang, Q. Gao, X. Li, Peroxymonosulfate activation by Co-doped magnetic Mn3O4 for degradation of oxytetracycline in water, Environmental Science and Pollution Research 29(26) (2022) 39249-39265.

[3] Y. Luo, Z. Liu, M. Ye, Y. Zhou, R. Su, S. Huang, Y. Chen, X. Dai, Synergistic enhancement of oxytetracycline hydrochloride removal by UV/ZIF-67 (Co)-activated peroxymonosulfate, Water 16(18) (2024) 2586.

[4] W. Huang, X. Jin, Q. Li, Y. Wang, D. Huang, S. Fan, J. Yan, Y. Huang, D. Astruc, X. Liu, Co_3O_4 nanocubes for degradation of oxytetracycline in wastewater via peroxymonosulfate activation, ACS Applied Nano Materials 6(13) (2023) 12497-12506.

[5] T. Deng, H. He, L. Zeng, H. Wang, Q. Zou, X. Gong, M. Sun, Y. Liu, J. Zhao, Oxygen-vacancies rich $CuFe_2O_4$ catalyst as efficient peroxymonosulfate activator for enhanced oxytetracycline degradation: Performance and mechanism, Chemical Engineering Science 291 (2024) 119945.

[6] X. Di, X. Zeng, T. Tang, D. Liu, Y. Shi, W. Wang, Z. Liu, L. Jin, X. Ji, X. Shao, Non-radical activation of peroxymonosulfate by modified activated carbon for efficient degradation of oxytetracycline: Mechanisms and applications, Separation and Purification Technology 349 (2024) 127877.

[7] N. Li, H. Li, C. Xu, Z. Zhou, T. Rao, R. Ji, S. Lin, J. Du, S. Xu, S. Lyu, Synergistic enhanced activation of peroxymonosulfate by heterojunction Co_3O_4–CuO@CN for removal of oxytetracycline: Performance, mechanism, and stability, Environmental Research 234 (2023) 116517.

[8] W. Mao, D. Wang, X. Wang, X. Hu, F. Gao, Z. Su, Efficient cobalt-based metal-organic framework derived magnetic Co@C-600 Nanoreactor for peroxymonosulfate activation and oxytetracycline degradation, Colloids and Surfaces

A: Physicochemical and Engineering Aspects 648 (2022) 129234.
